# Metaplastic carcinoma of the breast: real-world outcome from a tertiary cancer centre in India

**DOI:** 10.3332/ecancer.2022.1429

**Published:** 2022-07-14

**Authors:** Ananthi Balasubramanian, Priya Iyer, Rama Ranganathan, Kanchan Murhekar, Manikandan Dhanushkodi, Selvaluxmy Ganesarajah, Sridevi Velusami, Arvind Krishnamurthy

**Affiliations:** 1 Department of Radiation Oncology, Cancer Institute (WIA), Chennai 600 036, India; 2 Department of Epidemiology and Cancer Registry, Cancer Institute (WIA), Chennai 600 036, India; 3 Department of Oncopathology, Cancer Institute (WIA), Chennai 600 036, India; 4 Department of Medical Oncology, Cancer Institute (WIA), Chennai 600 036, India; 5 Department of Surgical Oncology, Cancer Institute (WIA), Chennai 600 036, India; a https://orcid.org/0000-0001-7107-8145

**Keywords:** metaplastic carcinoma, chemoresistance, receptor negative, triple negative, node-positive

## Abstract

Metaplastic carcinoma (MPC) is a rare subgroup of breast tumours accounting for <5% of all invasive breast cancers. Histologically confirmed 40 MPC from January 2001 to December 2018 were identified from our electronic database: stage I 2.5% (*n* = 1), stage II 40% (*n* = 16), stage III 45% (*n* = 18) and stage IV 12.5% (*n* = 5). The mean tumour size was 6 cm, node-negative in 60%, and hormone receptor-negative in 75%. Among the 35 non-metastatic patients, 17 (48.6%) received initial neoadjuvant treatment (NAT), followed by surgery, and only 1 had a complete pathological response. At a median follow-up of 60 months, 17% (*n* = 6) had a recurrence. All six of them had lung metastasis. The 5-year overall survival (OS) and disease-free survival were 64.4% and 66.3%, respectively. Age more than 46 years (*p* = 0.027), tumour size more than 5 cm (*p* = 0.037), and nodal positivity (*p* = 0.001) were predictors of OS. In node-positive patients, the 5-year OS in those who underwent initial surgery was 80% and after NAT was 21.4% (*p* = 0.069). In node-negative patients, the 5-year OS after initial surgery was 83.3% and after NAT was 90% (*p* = 0.380). A statistical significance could not be demonstrated due to the small number of patients. Due to chemoresistance, the concept of initial NAT in MPC of the breast is a subject to be studied in the future. Upfront surgery should be considered for operable diseases (including stage III), followed by a decision on adjuvant therapy. Optimal treatment and effective systemic therapy regimens are yet to be defined.

## Introduction

Metaplastic carcinoma (MPC) is a rare subgroup of breast tumours accounting for <5% of all invasive breast cancers. MPC consists of a group of heterogeneous elements. The neoplastic epithelium is differentiated into squamous cells and/or mesenchymal-looking elements. According to the World Health Organisation classification of breast tumours 2012 [1], MPC is classified as a low-grade adenosquamous carcinoma, fibromatosis-like MPC, squamous cell carcinoma, spindle cell carcinoma and carcinoma with mesenchymal differentiation. Carcinoma with mesenchymal differentiation includes chondroid, osseous, rhabdomyoid and even neuroglial differentiation with carcinomatous areas. Usually, a mixture of different elements is demonstrated in MPC with or without ductal carcinoma. Most of them are triple-negative, and the chemotherapy regimens used to treat MPC are the same as invasive breast cancer. In MPC, the standard protocols have different responses. They are associated with a poor prognosis [2–4] due to their chemoresistance [5–7] when compared with invasive ductal carcinoma (IDC) or triple-negative ductal carcinoma [8, 9]. These tumours tend to relapse in the lungs and central nervous system. Whether MPC has a worse prognosis than IDC is yet to be defined, and effective chemotherapy regimens in this subtype are the need of the hour. We conducted this retrospective study to determine the patient and tumour characteristics, response to standard treatment regimens, patterns of recurrence and survival outcomes in MPC over 17 years from a single institution.

## Materials and methods

The Institutional Ethics Committee of Cancer Institute (WIA), India, approved this study (Ethics Committee approval number IEC/2021/March 02). Women (age >18 years) diagnosed with metaplastic breast cancer from January 2001 to December 2018 were included in this analysis. The clinical data and treatment details were retrieved from the electronic medical record (EMR) by coding the diagnosis as metaplastic breast cancer. Patients whose treatment details were not available in the EMR were collected from the patients’ case records. Those who defaulted for follow-up were contacted by phone, and information regarding their status was obtained. The details included for demographic analysis were age, initial diagnostic procedure, histopathological subtype, clinical stage, including hormone receptor status, and HER2/neu status. The data captured included details of treatment received, including surgery, nodal involvement, chemotherapy, and radiotherapy details, including response (both clinical and pathological) to neoadjuvant treatment (NAT), details of recurrence as local, systemic or both and survival. Hormone receptor positivity (estrogen receptor (ER) and progesterone receptor (PR)) was defined as any score of 1% and above. HER2/neu positivity was taken as immunohistochemistry 3+ or if fluorescence *in-situ* hybridisation (FISH) was positive. All patients were treated after discussion in a multidisciplinary tumour board. Early stage (stages I and II) underwent surgery, followed by a decision on adjuvant therapy. Locally advanced stage (stage III) underwent NAT and was reassessed for surgery. Advanced metastatic stage (stage IV) received palliative therapy.

### Statistical analysis

All data were analysed with IBM SPSS statistics, version 22 (SPSS Inc., Chicago, IL). Demographic and clinicopathologic features were summarised by using descriptive statistics. Overall survival (OS) was defined as the time from diagnosis to death due to any cause or last follow-up date. Disease-free survival (DFS) was defined as the time from diagnosis to recurrence (local, distant or both) or breast cancer-related event or death. Time to recurrence was defined as the time from diagnosis to the first recurrence. Progression-free survival (PFS) was defined as the time from the date of recurrence in non-metastatic patients or from diagnosis in metastatic patients until death or the date of censoring. The objective response rate (ORR) was defined as the ratio of partial and complete responses to treatment. Survival was estimated by Kaplan–Meier’s method, and log-rank test was used to compare prognostic factors. All statistical tests were two-sided, and a *p*-value of <0.05 was considered significant.

## Results

Around 12,127 patients were diagnosed with breast cancer from January 2001 to December 2018 at Cancer Institute (WIA), Chennai, India. Among them, 40 patients (<1%) with metaplastic breast cancer were included in this analysis. Out of the 40, non-metastatic patients were 35 and metastatic patients were 5. The tumour, patient and treatment characteristics are shown in Table 1. The median age at presentation was 47 years (32–90 years). The mean size of the tumour was 6 cm (range = 2–16 cm). Majority of the patients presented with stage III (45%), followed by stage II (40%). Most of them were T2 (40%), T3 (27.5%) and T4 (27.5%). The lymph node status was positive in 40%. At presentation, five patients (12.5%) were diagnosed to have stage IV, with metastasis to lungs (*n* = 3), bone (*n* = 1) and brain (*n* = 1). The median follow-up duration was 60 months (5–161 months). The common pathological variant was mesenchymal differentiation in 21 (52.5%) and was also the common variant in ≤46 years (*p* = 0.03). The most common pathological diagnostic method was trucut biopsy in 65% and excision biopsy in 35%. Hormone receptors (ER and PR) were negative in 75% and triple negative in 37.5%.

Out of the 35 non-metastatic patients, 51.4% (*n* = 18) underwent upfront surgery, followed by adjuvant treatments, compared to 48.6% (*n* = 17) who received NAT. None of the HER2/neu-positive patients (*n* = 4) received trastuzumab. The responses observed to NAT (*n* = 17) were complete pathological response in one (5.9%), partial response in four (23.5%), static response in nine (53%) and progressive disease in three (17.6%). Out of the 35 non-metastatic patients, 91.4% (*n* = 32) underwent surgery, i.e., modified radical mastectomy in 30 and breast conservation surgery in 2, and 8.6% (*n* = 3) did not undergo surgery. Radiotherapy was administered in 68.6% (*n* = 24). In stage IV, one patient underwent a palliative mastectomy.

### Failure patterns and survival

For non-metastatic patients (*n* = 35), the median duration of follow-up was 71 months. Between 8 and 50 months, 17% developed systemic recurrences (*n* = 6). All six had lung metastasis (Table 2). Three of those who received NAT did not undergo planned surgery. During NAT, one had brain metastasis and the other two had local progressive disease only. One of the patients with local progressive disease considered inoperable had an initial biopsy of infiltrating breast carcinoma. A repeat biopsy was done because of local progression after NAT, which confirmed squamous cell carcinoma. The other patient was elderly on hormones and was inoperable due to local progressive disease. The median time to recurrence/progression was 15 months. The estimated OS at 5 years was 74.9% and DFS was 76%.

For metastatic patients (*n* = 5), the median follow-up duration was 9 months (5–26 months), with lung metastasis in 60% (*n* = 3), brain metastasis in 20% (*n* = 1) and bone metastasis in 20% (*n* = 1). The median PFS was 4 months (3–21 months), and none were alive at the end of 24 months. All received palliative therapy. One patient with an initial biopsy of triple-negative invasive breast cancer had local disease progression with the tumour size reaching 25 cm, and no response at the metastatic site (lungs), despite multiple lines of chemotherapy and palliative mastectomy was performed. The final diagnosis was a chondromyxoid variant of MPC and node-negative.

For the entire group of metaplastic patients (*n* = 40), at the time of censoring (December 2018), 15 patients had died (*n* = 15). Five were stage IV at presentation and died due to progressive disease. Out of the 10 non-metastatic patients, 6 died due to recurrent disease in the lungs. Out of the three patients who died during NAT, one died due to brain metastasis and two died due to progressive local disease. One died due to old age. The most common metastatic site was the lungs in 22.5%, brain in 5% and bone in 2.5%. The median PFS was 6 months. The 5-year OS and DFS were 64.4% and 66.3%, respectively.

### Predictors of survival among the studied metaplastic patients (*n* = 40)

**Figure 1.**
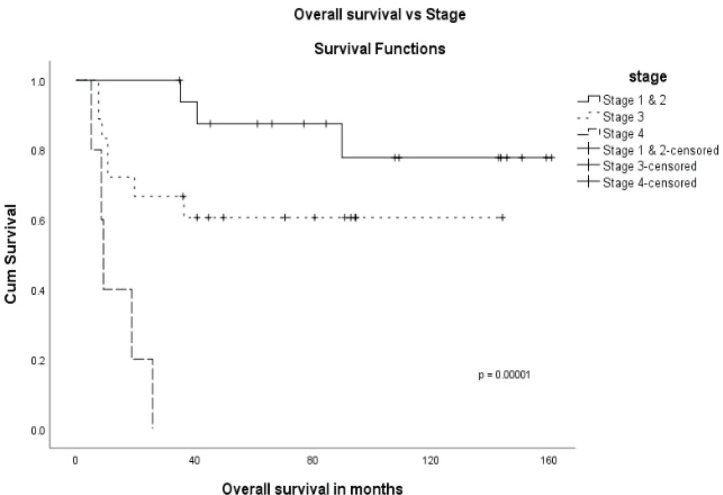
OS versus stage.

**Figure 2.**
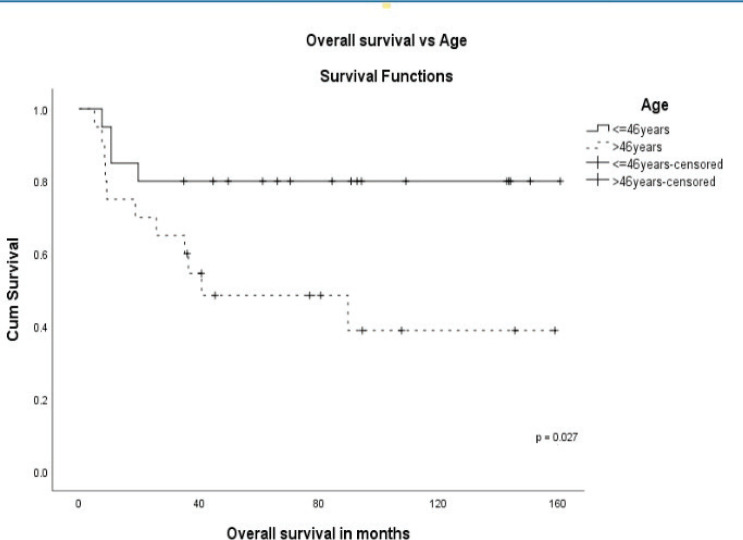
OS versus age.

**Figure 3.**
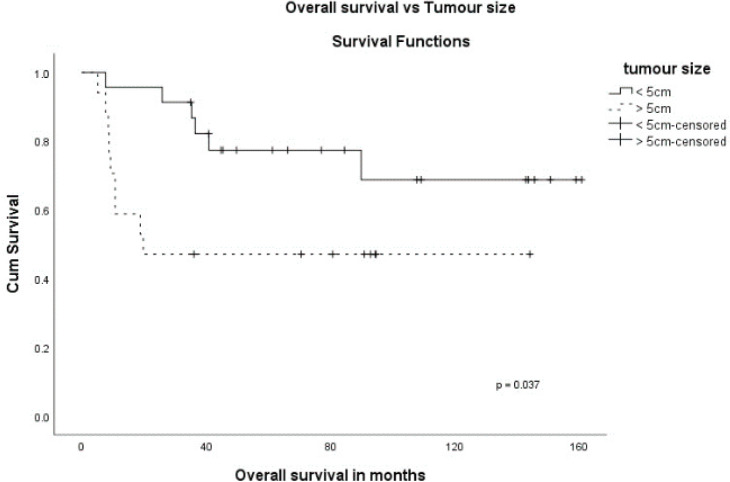
OS versus tumour size.

**Figure 4.**
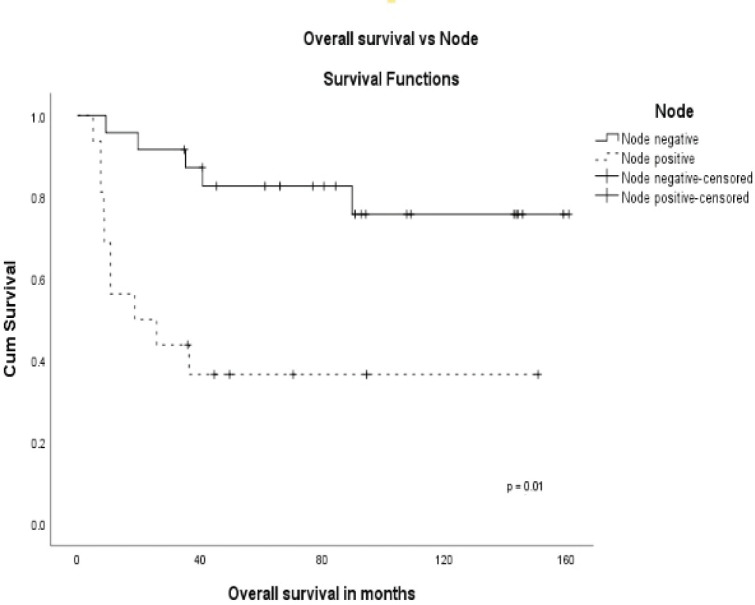
OS versus node.

The stage was a predictor of OS. The 5-year OS in stages I and II, stage III and stage IV was 87.5%, 60.6% and 0%, respectively (*p* = 0.00001) (Figure 1). Age more than 46 years (*p* = 0.027) (Figure 2), tumour size more than 5 cm (*p* = 0.037) (Figure 3) and nodal positivity (*p* = 0.001) (Figure 4) were predictors of OS.

### Predictors of survival in the non-metastatic patients (*n* = 35)

**Figure 5.**
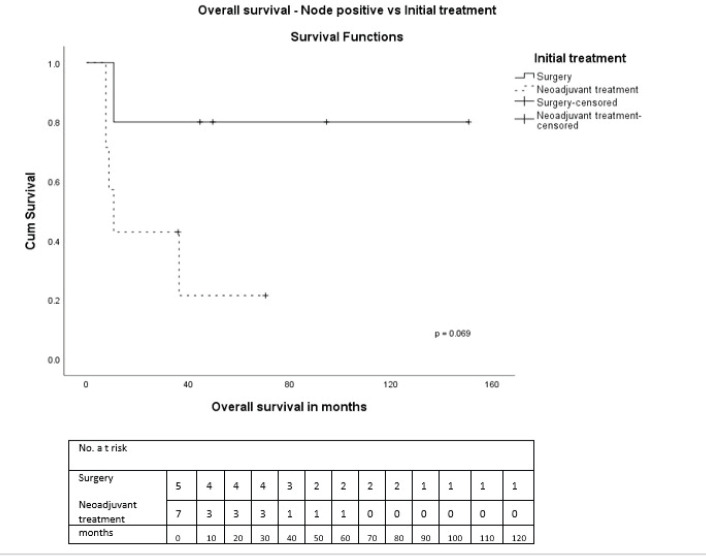
OS—Node-positive versus initial treatment.

**Figure 6.**
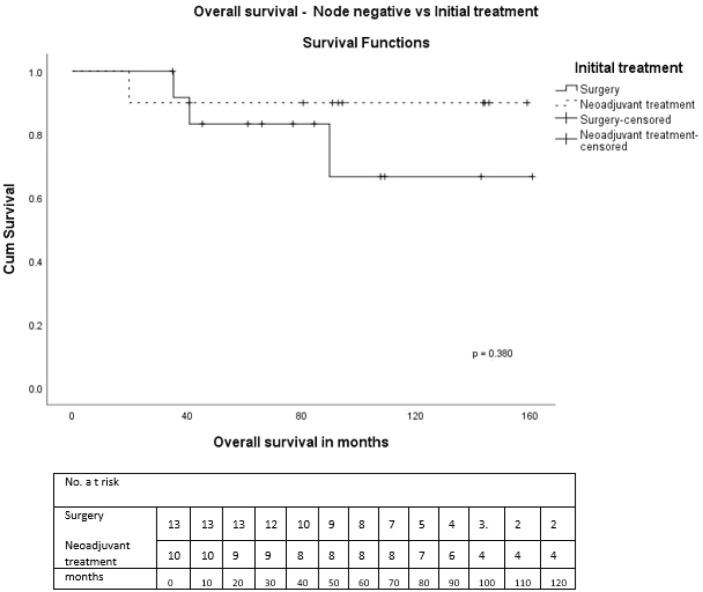
OS—Node-negative versus initial treatment.

On univariate analysis, there were no statistically significant differences in OS or DFS regarding age, tumour size, receptor status, pathological subtype, and treatments received. During NAT, three had progressive disease. Clinical stage III (*p* = 0.015), nodal positivity (*p* = 0.001) and chemotherapy in early stage (*p* = 0.018) were significant predictors for DFS, and nodal positivity (*p* = 0.013) for OS (Table 3). Also, both treatment modalities, i.e., either initial surgery or after NAT, in node-positive and negative patients were analysed. In node-positive patients, the 5-year OS in those who underwent initial surgery was 80% and after NAT was 21.4% (*p* = 0.069) (Figure 5). The 5-year OS after initial surgery was 83.3% in node-negative and after NAT was 90% (*p* = 0.380) (Figure 6). In node-positive MPT, NAT did not improve DFS or OS, demonstrating that in node-positive patients, NAT does not add to survival benefit due to chemoresistance and variable response to NAT. A statistical significance could not be demonstrated due to the small number of patients.

## Discussion

MPC is a rare but aggressive subtype of breast cancer [1–3]. MPC can coexist with ductal carcinoma in-situ (DCIS) or invasive mammary carcinoma. MPC presents with less frequent axillary nodal metastasis compared to IDC [4, 5, 8, 10, 11]. A clinicopathological analysis of 45 patients has reported axillary nodal involvement in 24%, and in some single institutional series, the reported incidence was 24%–28% [9–12]. In our study, axillary nodal involvement was observed in 40%. Also, MPC is more likely to have metastatic disease at presentation [4, 5, 8–10]. In our study, metastasis at presentation was reported in 12.5%. The majority of MPC were triple negative [1, 4, 6, 8, 13–15] and had a worse prognosis than non-metastatic triple-negative breast cancer (TNBC) [1, 4, 6, 8, 16]. Pezzi *et al*. [17] analysed 892 MPC and compared it with IDC and concluded that MPC presented with fewer T1 tumours (29% versus 65%), more of N0 (78% versus 66%) and fewer estrogen receptor-positive tumours (11% versus 74%). In the literature, HER2/neu expression (score 3+) was reported in 0%–25% [5, 7, 8, 17, 18]. We had 37.5% (*n* = 15) who were triple negative, receptor-negative in 75% (*n* = 30), receptor-positive in 25% (*n* = 10), HER2/neu positive in 10% (*n* = 4) and HER2/neu unknown status in 42.5% (*n* = 17). Also, they present with larger tumour sizes (T2, T3) [7–9, 15, 16, 19, 20] and are usually more aggressive than invasive mammary carcinoma [8, 21]. There is no specific characteristic finding in sonography and mammography. Some of the lesions are well defined, solid, cystic and can show benign characteristics [12, 22]. MPC is not a contraindication for breast conservation surgery (BCS), and the survival between BCS and mastectomy showed no difference [20, 23]. Given the larger sizes of tumours reported, most of the patients underwent a mastectomy. In our study group, two patients underwent BCS. Others underwent mastectomy due to the large size of the tumour, and 35% presented after an excision biopsy carried out at a clinic outside the institution. Sang Y *et al.* [8] reported a 5-year OS of 54.5% in MPC versus 85.1% in IDC and 73.3% in TNBC (*p* < 0.0001). A multicentric study of 405 patients reported no difference in OS between IDC versus MPC, and spindle cell type showed aggressive behaviour. In our study, out of the seven with spindle cell pathology, two (29%) were metastatic at presentation (Table 1) and three (43%) had recurrences (Table 2) during follow-up, confirming the aggressive nature of spindle cell type. In the early stage, chemotherapy improved survival [24]. In our study, in early-stage 5-year survival in those receiving chemotherapy was 86.7%. The local recurrence rate in large tumours is 35%–62% in the first 2–5 years compared to 17%–20% for IDC of the same tumour size. In our subset, the 5-year OS in tumours >5 cm was 47.1% versus 77.3% in ≤5 cm (*p* = 0.037). In one of the largest single-institution studies [11], 45 patients with MPC have reported a 5-year OS of 69% at a median follow-up of 28 months (range = 2–138 months). In our series, the 5-year OS was 64.4% at a median follow-up of 60 months (range = 5–161 months).

He *et al*. [25], in a retrospective population-based study of 1,112 metaplastic cancers, have shown tumours of larger size and less lymph node involvement consistent with our study. Also, they concluded that MPC had a worse prognosis than non-metaplastic triple-negative breast cancer and chemotherapy was not associated with improved survival. Also, as lymph node involvement is less likely, most patients underwent surgical options upfront and received less chemotherapy or radiotherapy than other TNBCs. One meta-analysis confirmed that adjuvant treatment after surgical management may improve 5-year OS in patients with MPC [26]. Our study also shows that NAT did not add to the OS benefit in the node-positive subset. Our analysis also confirms the inadequate response to NAT (Table 1) and initial surgical option in all operable non-metastatic patients should be considered.

The limitation of our study is the retrospective nature and small number. In our study, the tumours were almost equally staged in clinical stages I and II (*n* = 17) and stage III (*n* = 18). Also, irrespective of the tumour’s size, it was observed that 60% had negative axillary nodes, and most of them were receptor-negative (75%). An equal number of patients had undergone initial surgery or NAT, followed by surgery. However, as expected in receptor-negative and triple-negative tumours, the response to neoadjuvant chemotherapy did not increase complete pathological rates as expected. Only one patient had a complete pathological response, and 7 (41%) out of 17 who received NAT continued to be node-positive. Also, the lungs was the common site of recurrence within 15 months in those who had completed planned treatments, explaining the aggressive and chemoresistant nature of the disease (Table 2). Another patient also had brain metastasis during NAT and was considered inoperable. Whether upfront surgery in operable patients will improve survival is a subject to be studied in the future.

The limitation of our study is the retrospective nature and small number. In our study, the tumours were almost equally staged in clinical stages I and II (*n* = 17) and stage III (*n* = 18). Also, irrespective of the tumour’s size, it was observed that 60% had negative axillary nodes, and most of them were receptor-negative (75%). An equal number of patients had undergone initial surgery or NAT, followed by surgery. However, as expected in receptor-negative and triple-negative tumours, the response to neoadjuvant chemotherapy did not increase complete pathological rates as expected. Only one patient had a complete pathological response, and 7 (41%) out of 17 who received NAT continued to be node-positive. Also, the lungs was the common site of recurrence within 15 months in those who had completed planned treatments, explaining the aggressive and chemoresistant nature of the disease (Table 2). Another patient also had brain metastasis during NAT and was considered inoperable. Whether upfront surgery in operable patients will improve survival is a subject to be studied in the future.

## Conclusion

MPC is a chemoresistant tumour (ORR 31%) with a 5-year survival of 64%. NAT did not provide a survival benefit in node-positive subjects. These tumours have no significant response to chemotherapy, and there is a high chance of local disease progression and metastasis, making future surgical options after chemotherapy difficult. Upfront surgery should be considered for operable diseases (including stage III), followed by a decision on adjuvant therapy. Further studies are warranted to find the optimal treatment, and effective systemic therapy regimens are yet to be defined in MPC.

## Conflicts of interest

The authors have no conflicts of interest to report.

## Funding statement

The authors received no financial support for the research, authorship, and/or publication of this article.

## Figures and Tables

**Table 1.** Clinicopathological characteristics of the 40 patients with MPC.

Variable	Non-metastatic *n* = 35 (87.5%)	Metastatic *n* = 5 (12.5%)	Total *n* = 40 (%)
Age (median 47 years, range 32–90 years) ≤46 years >46 years	20 (57.1) 15 (42.9)	0 5 (100)	20 (50) 20 (50)
Tumour size T1 T2 T3 T4	02 (5.7) 15 (42.9) 11 (31.4) 07 (20)	0 1 (20) 0 4 (80)	02 (5) 16 (40) 11 (27.5) 11 (27.5)
Lymph node N0 N1 N2 N3	24 (68.6) 06 (17.1) 04 (11.4) 01 (2.9)	0 3 (60) 2 (40) 0	24 (60) 09 (22.5) 06 (15) 01 (2.5)
Initial stage Stage I Stage II Stage III Stage IV	01 (2.9) 16 (45.7) 18 (51.4)	IV = 5	01 (2.5) 16 (40) 18 (45) 05 (12.5)
Nodal status Negative Positive	23 (65.7) 12 (34.3)	1 (20) 4 (80)	24 (60) 16 (40)
Receptor status Negative (ER and PR) Positive (ER and/or PR)	26 (74.3) 09 (25.7)	4 (80) 1 (20)	30 (75) 10 (25)
HER2/neu status Negative (0) Positive (3+) Unknown	16 (45.7) 04 (11.4) 15 (42.9)	3 (60) 0 2 (40)	19 (47.5) 04 (10) 17 (42.5)
Pathological subtype Mesenchymal differentiation Fibromatosis like Squamous cell carcinoma Spindle cell type Adenosquamous	19 (54.2) 01 (2.9) 07 (20) 05 (14.3) 03 (8.6)	2 (40) 0 0 2 (40) 1 (20)	21 (52.5) 01 (2.5) 07 (17.5) 07 (17.5) 04 (10)
Initial treatment Surgery NAT^a^ Palliative chemotherapy Palliative radiotherapy	18 (51.4) 17 (48.6) 0 0	0 0 4 (80) 1 (20)	18 (45) 17 (42.5) 04 (10) 01 (2.5)
Chemotherapy^b^ CMF FEC AC + T No chemo	02 (5.7) 19 (54.3) 12 (34.3) 02 (5.7)	0 0 5 (100) 0	02 (5) 19 (47.5) 17 (42.5) 02 (5)
Response to NAT Partial response Complete response Static response Progressive disease^c^	04 (23.5) 01 (5.9) 09 (53) 03 (17.6)		
Surgery (Loco-regional) Done Not done	32 (91.4) 03 (8.6)	1 (20) 4 (80)	33 (82.5) 07 (17.5)
Radiotherapy (Loco-regional) Neoadjuvant Adjuvant Not given	12 (34.3) 12 (34.3) 11 (31.4)		
Metastasis / recurrence/progression Lung Brain Bone Local recurrence^d^ Local progression	06 (17.1) 01 (2.9) 0 0 02 (5.7)	3 (60) 1 (20) 1 (20)	09 (22.5) 02 (5) 01 (2.5) 0 02 (5)
DFS 3 years 5 years	79.3% 76.0%	Median PFS^¶^ = 4 months (3–21 months)	69.2% 66.3%
OS 2 years 3 years 5 years	82.9% 76.8% 74.9%	20% 0 0	75% 70% 64.4%

a NAT—Neoadjuvant treatment ((chemotherapy ± radiotherapy) (*n* = 17/35)

b FEC: 5-Fluorouracil, Epirubicin, Cyclophosphamide

CMF: Cyclophosphamide, Methotrexate,5-Fluorouracil

AC+T: Adriamycin, Cyclophosphamide+Paclitaxel

c Progressive disease in three non-metastatic cases (one had brain metastasis and two had local progressive disease)

d Two had local recurrence and one had soft tissue thigh metastasis in addition to lung metastasis—included in lung metastasis

¶ PFS: Progression-free survival

**Table 2.** Details of recurrent MPC.

Recurrent MPC *n* = 6	1	2	3	4	5	6
Age (in years)	46	45	64	45	41	63
Clinical stage	3	3	3	3	3	1
Pathological type	MD	SCC	Spindle	Spindle	Spindle	SCC
Nodal status	Positive	Negative	Positive	Positive	Positive	Negative
Initial treatment	Surgery	NAT-surgery	NAT-surgery	NAT-surgery	Surgery	Surgery
Receptor	Negative	Negative	Negative	Positive	Negative	Negative
Response to NAT	Not applicable	Static	Node negative	Node positive	Not applicable	Not applicable
Site of recurrence	Local + lung	Lung	Lung	Lung	Local + lung	Lung
Time to recurrence (in months)^a^	10	17	36	10	31	38

MPC: Metaplastic carcinoma; MD: Mesenchymal differentiation; SCC: Squamous cell carcinoma; NAT: Neoadjuvant treatment

a Time from the first diagnosis to recurrence

**Table 3.** Univariate analysis of prognostic variables in non-metastatic MPC.

Variable	5-year DFS (%)	*p*-value (log rank)	5-year OS (%)	*p*-value (log rank)
Age ≤46 years >46 years	75.0 56.8	0.280	80.0 64.6	0.230
Initial stage Stages I and II Stage III	93.3 59.3	0.015	87.5 60.6	0.103
Nodal status Negative Positive	90.9 46.3	**0.001**	86.3 48.6	**0.013**
Receptor status Negative (ER and PR) Positive (ER and/or PR)	72.0 87.5	0.401	71.9 77.8	0.670
Pathological subtype Mesenchymal differentiation Fibromatosis like Squamous cell carcinoma Spindle cell type Adenosquamous	89.5 100 51.4 30.0 100	0.062	89.5 100 42.9 40.0 100	0.068
First treatment Surgery followed by adjuvant therapy NAT followed by surgery	82.5 69.3	0.298	82.6 64.2	0.323
Chemotherapy Stages I and II Stage III	92.9 58.8	**0.018**	86.7 64.2	0.102
Radiotherapy Not given Given	90.0 69.3	0.154	74.0 73.4	0.744
Node-positive Surgery followed by adjuvant therapy NAT followed by surgery	60.0 35.7	0.354	80.0 21.4	0.069
Node-negative Surgery followed by adjuvant therapy NAT followed by surgery	90.9 90.0	0.870	83.3 90.0	0.380
